# Perceptions of health care providers and patients on quality of care in maternal and neonatal health in fourteen Bangladesh government healthcare facilities: a mixed-method study

**DOI:** 10.1186/s12913-015-0918-9

**Published:** 2015-06-19

**Authors:** Farzana Islam, Aminur Rahman, Abdul Halim, Charli Eriksson, Fazlur Rahman, Koustuv Dalal

**Affiliations:** School of Health and Medical Sciences, Örebro University, Örebro, Sweden; Centre for Injury Prevention and Research, Bangladesh (CIPRB), House: B-162, Road 23, Mohakhali DOHS, Dhaka 1206 Bangladesh

**Keywords:** Quality of healthcare, Maternal and neonatal health, Healthcare providers’ perception, Clients’ satisfaction, Bangladesh

## Abstract

**Background:**

Bangladesh has achieved remarkable progress in healthcare with a steady decline in maternal and under-5 child mortality rates in efforts to achieve Millennium Development Goals 4 and 5. However, the mortality rates are still very high compared with high-income countries. The quality of healthcare needs improve to reduce mortality rates further. It is essential to investigate the current quality of healthcare before implementing any interventions. The study was conducted to explore the perception of healthcare providers about the quality of maternal and neonatal health (MNH) care. The study also investigated patient satisfaction with the MNH care received from district and sub-district hospitals.

**Methodology:**

Both qualitative and quantitative methods were used in the study. Two district and 12 sub-district hospitals in Thakurgaon and Jamalpur in Bangladesh were the study settings. Fourteen group discussions and 56 in-depth interviews were conducted among the healthcare providers. Client exit interviews were conducted with 112 patients and their attendants from maternity, labor, and neonatal wards before being discharged from the hospitals. Eight physicians and four anthropologists collected data between November and December 2011 using pretested guidelines.

**Results:**

The hospital staff identified several key factors that affected the quality of patient care: shortage of staff and logistics; lack of laboratory support; under use of patient-management protocols; a lack of training; and insufficient supervision. Doctors were unable to provide optimal care because of the high volume of patients. The exit interviews revealed that 85 % of respondents were satisfied with the hospital services received. Seven out of 14 respondents were satisfied with the cleanliness of the hospital facilities. More than half of the respondents were satisfied with the drugs they received. In half of the facilities, patients did not get an opportunity to ask the healthcare providers questions about their health conditions and treatments.

**Conclusion:**

The quality of healthcare is poor in district and sub-district hospitals in Bangladesh because of the lack of healthcare personnel and logistic support. An integrated quality improvement approach is needed to improve MNH care service in district and sub-district hospitals in Bangladesh.

## Background

The World Health Organization (WHO) estimates that nearly 289,000 women died of complications during pregnancy or childbirth in 2013, and among them, 99 % of all maternal deaths occurred in the developing countries of Sub-Saharan Africa and South Asia [1]. In 2013, one third of global maternal deaths occurred in India (17 %) and Nigeria (14 %) [2]. There are nearly three million neonatal deaths worldwide every year, with two thirds in ten Asian and African countries. In India and Nigeria, neonatal mortality is high, which is similar to the high number of maternal deaths [3]. As a developing country, Bangladesh has made key achievements in both maternal and neonatal health (MNH) during the last decade. In Bangladesh, the maternal mortality ratio declined from 322to 170 per 100,000 live births between 2001 and 2013 and neonatal mortality ratio declined from 52 to 24 per 1000 live births between 1994 and 2013 [2, 4–6]. However, Bangladesh still needs to make further efforts to decrease the maternal and neonatal death rates to achieve Millennium Development Goals (MDGs) 4 and 5.

Maternal and neonatal deaths are usually associated with a poor health environment and a serious lack of healthcare resources, including medicines, healthcare professionals, and healthcare infrastructure [7, 8]. Poor quality care during hospital births is a major contributing factor to maternal- and neonatal-related complications in developing countries [9–11].

In the developing world, including African countries, healthcare systems suffer from various inadequacies related to staff training, prenatal screening, knowledge and use of evidence-based protocols, prompt cesarean delivery, multidisciplinary care, and lack of quality improvement (QI) support [12]. A weak healthcare system places women and their babies at risk for morbidity and mortality.

High maternal and neonatal deaths and stillbirth rates along with the inadequacies and dissatisfaction of healthcare providers and recipients indicate that the quality of healthcare is poor in Bangladesh [8]. An evaluation of the Health Nutrition and Population Sector Program by the government of Bangladesh (GoB) raised quality issues as priority gap to resolve in service provision in healthcare delivery, particularly in MNH. Considering this issue, the Bangladesh Ministry of Health and Family Welfare has taken the appropriate steps to improve quality of care as a priority in MNH [13]. To accelerate progress in achieving MDGs 4 and 5 in recent years, the GoB has been implementing the Joint GoB–United Nations MNH Initiative (MNHI) program in four districts of the country, Narail, Jamalpur, Thakurgaon, and Moulvibazar. At the facility level, the MNHI program provides technical support to implement facility-based emergency obstetric care and neonatal healthcare services with a special focus on quality care through the development of human resources, strengthening the referral system, timely supply/procurement of logistics and drugs, regulating management information system (MIS) and linking it with the community, and organizing coordination and review meetings/workshops. It also mobilizes ample resources to encourage innovations and strengthen healthcare systems for better performance [14, 15]. Therefore, QI is central to progress in MNH services.

However, very few studies have been carried out to assess the quality of healthcare services in Bangladesh, including the MNH care provided by the public hospitals. This study was conducted to explore the perception of healthcare providers regarding the quality of their MNH care, as well as to investigate patients’ satisfaction with their MNH care from the district and sub-district hospitals in Bangladesh.

## Methods

Mixed-method approaches (both qualitative and quantitative) were adopted to collect data between November and December 2011. Specifically, group discussions (GDs) with healthcare administrators and professionals and in-depth interviews (IDIs) with paramedics and ancillary staff to explore healthcare providers’ perception on the quality of care. Client exit interviews were conducted with patients and their attendants to assess their satisfaction with the healthcare received at the facility.

Fourteen public hospitals in Thakurgaon and Jamalpur districts were purposively selected as the study settings based on assessment of progress towards achievement of MDGs 4 and 5. Thakurgaon was selected for high performance and Jamalpur for low performance [16].

The study settings were district-level hospitals, comprising two district hospitals and two maternal and child welfare centers (MCWCs), and ten sub-district-level hospitals called upazila health complexes (UHCs). UHCs are primary healthcare centers and the first point of referral. Each of these health complexes serves a population of between 200,000 and 400,000 and has a bed capacity of between 31 and 50.

### Qualitative methods

Participants for GDs and IDIs were selected purposively. The healthcare administrators and professionals at MNH services, including consultants, medical officers, nurses, and family welfare professionals, and visitors to three types of healthcare facilities were selected for GDs. The IDIs were conducted with paramedics and ancillary staff, including laboratory technicians, pharmacists, and maternal and neonatal ward supervisors and staff (Table 1). They were selected as “information-rich respondents” with extensive knowledge and experience in providing MNH services.

**Table 1:** Methodology matrix of the study

Study design	Methods & numbers	Research instruments	Respondents
Qualitative study	*GDs: 14 (One group from each hospital)	GD guideline: semi-structured open-ended questions with probes	From district hospitals: civil surgeon, obstetrics and pediatrics consultants, anesthesiologist, nursing superintendent, and two senior staff nurses
			From MCWCs: deputy director of family planning, anesthesiologist, medical officer, one nurse, and one family welfare visitor
			From UHCs: upazila health and family planning officer, obstetrics and gynecology specialists, pediatrician, anesthesiologist, resident medical officer, and one nurse
	**IDIs:56 (four interviews from each hospital)	IDI guideline: semi-structured open-ended questions with probes	From district hospitals, MCWCs, and UHCs: One laboratory technician/pharmacist, one ward master/nursing supervisor, one ancillary staff from the maternal or neonatal wards, and one cleaner
Quantitative study	Exit interviews: 112 (eight exit interviews from each hospital)	Structured questionnaire	Patients or their attendants from maternal, labor, and neonatal wards at district hospitals, MCWCs, and UHCs

*GD* group discussion, *IDI* in-depth interview, *MCWC* maternal and child welfare center, *UHC* upazila health complex

The GDs and IDIs with healthcare professionals explored the following aspects of MNH service provision: perceptions and experiences of MNH service provision, barriers to providing quality MNH services to the patients, and suggestions regarding how to improve the quality of MNH services.

In conducting GDs and IDIs, semi-structured open-ended questions with probes were used and pretested in one of the public hospitals not included in the study. The qualitative data was collected by a team comprised of two physicians and one anthropologist. Four teams were involved in the data-collection process and they received three days of training from the investigators before they conducted GDs and IDIs. The teams visited each hospital to collect relevant data. GDs and IDIs took 120–150 and 30–40 min to complete, respectively. The interviews were audio taped and handwritten notes were made. In each type of session, a facilitator requested one of the participants to express his/her perception about the MNH services provided to the patients from their healthcare facility. This was an ice-breaking exercise as well as a thought-provoking mechanism for the participants. The facilitator then gradually introduced a series of prompts to encourage discussion about their perception on the quality and availability of healthcare infrastructure, cleanliness, services, training, and adequate personnel and logistics, laboratory support, use of patient-management protocols, and the necessity of training and supervision.

The collected qualitative data was analyzed following a thematic approach. First, the audiotape record and handwritten notes were transcribed and then the transcripts were checked and validated. A thematic framework was developed with the coding of transcription undertaken by using this framework. Finally, the data was charted and interpreted.

### Quantitative methods

A convenient sampling method was implemented in the exit interviews to help recruit the respondents (mothers of newborns or their attendants) who had received services from maternal, labor, and neonatal wards at least 3 days following discharge (Table 1). Attendants were interviewed when patients could not participate in the interview because of their general discomfort or weakness, or when the mother of a newborn did not accompany their newborn at the hospital.

The data was collected by two physicians. Four teams were involved in the data-collection process. The teams visited each hospital and interviewed the patients discharged during that day or their attendants. A total of 112 exit interviews were conducted, with eight from each hospital.

For data collection, a structured questionnaire was developed through workshops attended by obstetricians, pediatricians, and relevant program personnel from the Bangladesh Ministry of Health and Family Welfare. The structured questionnaire was first developed in English and later translated into Bangla for the use of the respondents. To check its internal validity, the Bangla version was back-translated into English. The prepared questionnaires were pretested in a public hospital not included in the study. The pretested structured questionnaire included questions related to the patient waiting time to receive care, level of satisfaction with the cleanliness of the hospital, drug supplies, adequate time given by healthcare professionals and the opportunity to ask questions, and the MNH services provided to the patients. The data was entered into Epi-Info (Centers for Disease Control and Prevention, Atlanta, GA) and analyzed by both Epi-Info and SPSS (version 17; SPSS Inc, Chicago, IL). The mean age and percent distribution of the satisfaction level of the clients were calculated. The Chi-squared test for p value was calculated to obtain the significance of the differences in the various areas of satisfaction levels of the clients in different hospitals.

### Ethical issues

The study was approved by the Ethical Review Committee of the Centre for Injury Prevention and Research, Bangladesh. Informed consent was obtained from each of the participants involved in the IDIs and GDs. In conducting the exit interviews, written consent was obtained from the patients or their attendants. All three types of written consents were obtained after explaining to the participants about the purpose of the study and the importance of their views as service providers and clients.

For privacy and confidentiality of information, the study participants were reassured that all information received would remain anonymous and the collected data would be used for this research only. They were also informed that their participation would be voluntary and they could withdraw from the interview at any point.

Hard copies of the collected data were kept in a secured place. The interviewers and investigators had access to the audiotape data. The electronic databases were secured by setting a password and had access only to the investigators and the data manager involved in the study.

## Results

The results of this study have been described in two parts. The first part is qualitative, which covers the perception of healthcare providers and support staff on quality care. The second part covers the results of the quantitative method, where patients’ satisfaction on quality of care was assessed through exit interviews.

### Qualitative results

#### Perceptions of the healthcare providers: GDs and IDIs

GDs with the managers and senior-level professionals at healthcare service providers and IDIs with the lab technicians/pharmacists, ward masters/nursing supervisors, ancillary staff at the maternal or neonatal ward, and the cleaners of all 14 hospitals were organized in the Thakurgaon and Jamalpur district hospitals. All respondents in both types of interviews stated that the MNH services had rapidly expanded because of a MNHI program. At the facility level, the MNHI program provides technical support to implement facility-based emergency obstetric care and neonatal healthcare services with a special focus on quality care through the development of human resources, strengthening the referral system, timely supply/procurement of logistics and drugs, regulating MIS and linking it with the community, and organizing coordination and review meetings/workshops. It also mobilizes ample resources to encourage innovation and strengthen healthcare systems for better performance. However, the quality of care was still a great concern. The main issues affecting the quality of healthcare services revealed by the healthcare providers were: i) shortage of personnel, workload, and overcrowding, ii) inadequate logistics and laboratory support, iii) under use of patient-management protocols, iv) lack of training, and v) insufficient supervision.

#### Shortage of personnel, workload, and overcrowding

The GD and IDI data showed that most of the participants from all 14 facilities in both districts perceived that a shortage of personnel at different levels was the main barrier to ensure the quality of care in their facilities. The majority of the respondents mentioned that they were not very happy with the present situation of the quality of care in these hospitals. In terms of the volume of work, the respondents exceeded their existing capacities to deliver healthcare to their patients.

In a GD, one of the doctors from Jamalpur District Hospital said, “This is a 100-bed hospital, but we have around 250 patients. This is very difficult for us. On average, each doctor serves 40 patients a day.”

Regarding the shortage of hospital beds and infection control, during a GD one of the doctors from the neonatal ward of the Jamalpur District Hospital stated, “This is a 100-bed hospital, but there are always about 300 patients staying here. Two thirds of patients share a bed and naturally, it is very easy to transmit infections from one patient to another. Therefore, we are using antibiotics for almost all patients to prevent hospital-acquired infections.”

In another GD, the medicine specialist at Thakurgaon District Hospital described his experience as: “I need to work 96–100 h a week. It is really difficult for a person to work in this way for a long time and to maintain the expected quality of work.”

During a GD, a medical officer at Jamalpur MCWC said, “After delivery, when the mother and the baby are both in a bad condition, we are in great trouble. To whom should I attend, the mother or the baby?”

Similarly to respondents from the district hospitals and MCWCs, most of the respondents from the UHCs also reported the shortage of human resources in their facilities, which caused an overloading of their responsibilities.

In the IDIs, about half of the ward masters and nursing supervisors, and all of the ancillary staff of all surveyed healthcare facilities reported that because of the shortage of personnel they were overburdened with a heavy workload, which was similar to the findings of the GDs. As a result, they could not provide quality healthcare to their patients. All the cleaners interviewed suggested to take measures to restrict visitors because the wards often become messy and dirty because of overcrowding by visitors.

#### Inadequate logistics and laboratory support

Almost all of the healthcare providers at the 14 facilities stated that they lacked logistic support in the drug-dispensary, laboratory, and imaging services, and the blood bank. They also emphasized that their facilities did not have adequate infrastructure.

In a GD, a doctor from an UHC in Thakurgaon district said, “Our X-ray machine is not working. We have an incubator, but we do not have its operator. So it is useless…. There are only two doctors available in the hospital”.

The doctors and nurses at all the UHCs noted during their GDs that because of the lack of a blood bank, they could not manage their patients with ante- or postpartum hemorrhages and the quality of care could not be ensured. On the contrary, healthcare providers at the two MCWCs said that logistic support was available and they could maintain some standard of quality care. However, they expressed that there were opportunities to improve the quality of care further.

During IDIs, most of the ward masters and nursing supervisors said that because of an inadequate and irregular supply of logistics and medicines and the lack of a blood bank, it was difficult to manage regular healthcare activities. All laboratory technicians interviewed stated that they performed routine laboratory tests only. Although they had the expertise to perform other important laboratory tests, they could not perform these tests because of the unavailability of the required instruments and reagents.

#### Under use of patient-management protocols

Regarding the use of patient-management protocols, the majority of the respondents of GDs conducted in the district hospitals reported that they had protocols on eclampsia and diarrhea. However, it was not always possible for them to follow those protocols because of the shortage of personnel. One of the nurses from the Jamalpur District Hospital said, “We can’t always provide care according to the protocol due to shortage of personnel.”

However, most of the participants from the two MCWCs in the GDs expressed that they could follow the patient-management protocols to manage postpartum hemorrhage and eclampsia patients. Participants from most of the UHCs mentioned that they only used protocols to treat diarrhea and tuberculosis cases, and to provide postnatal and neonatal care. They opined that quality of care could be much better if they could use protocols for other health events.

#### Lack of training

Healthcare providers expressed their concern about not receiving any in-service training and refresher courses in obstetric and neonatal care, in particular in general and emergency management. They noted that several important and new management issues in obstetrics are emerging, in which they need to be well versed. This critically affects the quality of care rendered to the women and often results in loss of life. The providers’ dissatisfaction with a lack of training and refresher courses was expressed in a GD by one of the obstetricians at Thakurgaon District Hospital: “Since I qualified, I remember that I only attended an obstetrics life-saving skills training and a post abortion care course. But I cannot remember what we were taught; I need a refresher course”.

In response to the question regarding training, the majority of the IDI respondents, including nursing supervisors and laboratory technicians, mentioned that training could be a good initiative for an improved quality of care. The cleaners, who did not receive any sort of professional training, said that it would be an advantage for them to learn how to work better if they could have some training in future. According to their perceptions, in-service training needs to be organized regularly to enhance the quality of healthcare.

#### Insufficient supervision

Both doctors and nurses reported that there was no supervision of their clinical work by their higher authorities. They said that merely having management guidelines was not enough to maintain the quality of care, as they often did not have time to follow the guidelines during emergencies.

The following quote from a GD by a medical officer at Jamalpur District Hospital depicts the situation regarding supervision: “I feel there is no supervision at this hospital because when we are working in the labor ward, we work alone and we manage the wards by ourselves”.

In IDIs, when the participants were asked about monitoring and supervision, the majority of the respondents stated that the monitoring and supervision processes at their hospitals were weak. When they were asked if they would accept a system of accountability, all reported affirmatively. They expressed that a system of accountability would boost their morale to work more sincerely and with more responsibilities. They also considered that the system could stop unauthorized absences from the workplace.

#### Suggestions from healthcare providers on how to improve the quality of care

Healthcare providers from all the hospitals were willing to participate in a quality-assurance process if it really helps them to improve their working environment. The healthcare providers suggested that these QI issues should be discussed at a national level and solutions should come from there. In both GDs and IDIs, the respondents reported that training should be organized for all types of healthcare providers to help them maintain a standard of quality healthcare services. In addition, many participants proposed that extra incentives should be offered to the healthcare providers to motivate them to improve their quality of care. They also suggested that there should be a body, including service providers, nongovernmental organization partners, and political parties, responsible for the monitoring and supervision of all types of facility activities to ensure the quality of care.

### Quantitative results

#### Client satisfaction: exit interviews

In this study we assessed the clients’ (both patients and attendants of maternity, labor, and neonatal wards) level of satisfaction regarding the quality of care they received. The distribution of the clients or their attendants is provided in Table 2.

**Table 2:** Distribution of the samples by age for exit interviews

Ward	Type of interviewee	Age group			Subtotal	Total
		<18 years	18–40 years	>40 years		
Maternity/labor	Patient	6	53	5	78	112
	Attendant	0	8	6		
Neonatal	Mother of newborn	3	26	0	34	
	Attendant	0	3	2		

The mean age of the interviewees was 29.8 ± 10.7 years

Clients’ perception of satisfaction were judged in six areas: 1) waiting time to receive care from healthcare providers after entering the hospital; 2) cleanliness of the hospital; 3) adequate time given by the healthcare providers; 4) the opportunity to ask the healthcare providers questions about their care; 5) satisfaction with the drugs received from the hospitals; and 6) satisfaction with the MNH services received from the hospital.

From the exit interviews, it was revealed that the average mean waiting time before being seen by a healthcare provider was 10.95, 9.67, and 4.79 min at district hospitals, MCWCs, and UHCs, respectively.

The proportion of respondents who were satisfied with the cleanliness of the hospital was higher at UHCs (82.5 %) than at district hospitals and MCWCs (50.0 % and 43.7 %, respectively). The difference was statistically significant (*p* = 0.001). Regarding drug dispensing, 75.0 % and 56.2 % of the clients of the district hospitals and UHCs were satisfied; however, only 25.0 % of the respondents from the MCWCs were satisfied with the drugs they received (*p* = 0.015). More than three-fourths of the respondents of all three types of hospitals mentioned that physicians had given enough time to them. There was no statistical significant difference (*p* = 0.405) in the proportions of the respondents’ response on time given by the physicians among the three types of healthcare facilities. However, when the same clients were asked whether they had enough opportunities to ask questions to the physicians regarding their health issues, only 31.0 % of the respondents from the district hospitals replied positively and a small proportion of the clients of the MCWCs and UHCs (6.2 % and 10.0 %, respectively) had opportunities to ask questions. These differences were statistically significant (*p* = 0.045). More than 87.0 % of the clients of all three categories of hospitals were satisfied with the MNH services they received during their stay at hospital; however, no statistical difference was observed (*p* = 0.834) (Table 3).

**Table 3:** Distribution of clients who were satisfied in different domains of hospital healthcare in the selected hospitals

Area of satisfaction n (%)	Respondents by type of healthcare facilities (*N* = 112)			
	District hospitals (*n* = 16)	MCWC (*n* = 16)	UHCs (*n* = 80)	*P* value
Satisfied with cleanliness	8 (50.0)	7 (43.7)	66 (82.5)	0.001
Received drugs	12 (75.0)	4 (25.0)	45 (56.2)	0.015
Adequate time given by physician	12 (75.0)	13 (81.2)	70 (87.5)	0.405
Opportunity to ask questions	5 (31.0)	1 (6.2)	8 (10.0)	0.045
Satisfied with MNH services received	14 (87.5)	15 (93.7)	72 (90.0)	0.834

*MNH* maternal and neonatal health, *MCWC* maternal and child welfare center, *UHC* upazila health complex

## Discussion

This study was carried out to explore the perception of healthcare providers regarding the quality of MNH care, as well as to investigate patients’ satisfaction with the MNH care received from the district and sub-district hospitals in Bangladesh.

According to the perception of the healthcare providers of all three categories of hospitals, the quality of MNH services is poor because of the lack of healthcare personnel and logistic and laboratory support, as well as the under use of patient-management protocols, and a lack of training and supervision. However, the majority of the patients were satisfied with the care they received.

The infrastructure of public healthcare facilities covers the village level (for every 6,000 people, there is one community clinic). However, the quality of care remained debatable [17]. This study revealed that a shortage of personnel was the main barrier to ensuring the quality of care at different types of facilities. This shortage leads to work overload on the few available doctors, nurses, and support staff available, which further reduces morale and the ability to provide quality care. Our study findings regarding the association of quantity of human resources and quality of care are also in conformity with the findings of a Malawi study and a hospital survey conducted in 12 countries of Europe and in the United States among the nurses and patients on patient safety, satisfaction, and quality of hospital care [18, 19].

In this study, it was revealed that because of an inadequacy of beds and patient overcrowding, sometimes two or three patients share one bed and they have a higher chance to develop hospital-acquired infections and compromise quality care. Likewise, emergency departments are also overloaded with patients. The findings from a number of studies show that overcrowding in the inpatient and emergency departments contributes to poor quality of care [20–23].

In the current study, doctors and nurses acknowledged that the insufficient supply of laboratory support and an absence of blood-transfusion systems have an effect upon the quality of the diagnosis and treatment of the patients. A similar situation was revealed in two studies conducted in Bangladesh and Malawi [18, 24].

Furthermore, our study showed that doctors, nurses, and other healthcare professionals did not always use medical protocols to provide standardized care in these medical settings. However, written protocols are a prerequisite for standardized patient care [25].

Most of the doctors and nurses mentioned that they did not receive any formal training or refresher training after their graduation from medical colleges or nursing institutes, which promotes their lack of efficiency to provide quality of care. From an educational impact of a hospital-based neonatal resuscitation program in Ghana, it was revealed that practical evaluation of knowledge and skills improved from 58 % to 81 % after postgraduate training, and this improvement continued for 9–12 months after training [26]. In our study, according to the perception of healthcare professionals at different levels, adequate supervision is an important component to provide quality care, and a similar finding was found in a study conducted in Malawi in 2010 [18].

Although healthcare providers at all three categories of hospitals opined that they were unable to provide quality of care, most patients at the same hospitals were highly satisfied with the services they received. These two findings were self-contradictory.

Studies conducted in Bangladesh showed that the major contributing factors of being satisfied were short waiting times and a long consultation time with healthcare providers [27, 28]. In one of those studies, waiting times were found to be less than 11 min [28]. In our study, we also found similar short waiting times and longer consultation periods, and these might be the causes of patients’ satisfaction. In developing countries, the clients of the public hospitals mainly come from poor socioeconomic backgrounds with limited education and are not aware of their health rights [29]. Less-educated people are more satisfied in terms of the healthcare services that they receive [30]. Although we did not determine any socioeconomic factors by including the education level of the patients in our study, the reason for satisfaction could be the same. In general, the majority of poor people seek care from the public hospitals in Bangladesh [31]. Moreover, Bangladeshi expectations are quite low and they are content with minimum requirements, which were revealed by the World Happiness Survey 2009 conducted by the London School of Economics [32].

The mixed methodology matrix was the strength of this study, as throughout we tried to find perceptions from multiple perspectives to enhance and enrich the meaning of quality of care of the healthcare facilities. Here, qualitative and quantitative data were merged to develop a more complete understanding about the perception of both types of respondents, as well as compare and validate the surveyed data.

Some limitations exist in this study. First, the sampling of facilities was not randomized, but they included all the district and upazila hospitals in the two districts. However, there are 593 public health facilities in 64 districts of Bangladesh [33]. Because of a resource limitation, our research purposively selected hospitals from only two districts. Second, a convenient sampling method was followed in the exit interviews to recruit patients or their attendant, who had experienced healthcare in maternal and neonatal wards. Apart from age, no other socio-demographic information was collected from the exit interviewees. At the same time, the respondents might have given responses that they felt would please the healthcare providers rather than a true reflection of their feelings. In addition, the small number of study participants limits the generalizability of the results.

## Conclusion

The quality of MNH care is poor in district and sub-district hospitals in Bangladesh because of a lack of healthcare personnel and logistic support, including equipment, essential drugs, and laboratory needs. This information could be used to strengthen the national-level policy for improving the quality of MNH care at the facilities. In each type of public hospital in Bangladesh, there area fixed number of healthcare personnel and a fixed amount of logistic support, as specified by the GoB. However, the population of each district or sub-district is not the same. Therefore, a change in the policy is required to ensure the distribution of healthcare personnel and logistic support should be proportionate to population of the district or sub-district. In our study, it was also revealed that healthcare providers were dissatisfied with their quality of care; however, the majority of their patients were satisfied with their level of care. This is mainly because the patients were unaware of their health rights. An awareness-raising activity should be launched to educate patients that it is their right to obtain quality care.
